# Becoming a Sexual Assault Nurse Examiner

**DOI:** 10.1111/tct.70059

**Published:** 2025-03-08

**Authors:** Marivic B. Torregosa, Maria Del Rosario Benavides

**Affiliations:** ^1^ Texas A&M International University College of Nursing and Health Sciences 5201 University Boulevard Laredo Texas USA

**Keywords:** forensic nursing, medically underserved communities, qualitative research, SANE training, sexual assault nurse examiners

## Abstract

**Aim:**

There is a shortage of sexual assault nurse examiners (SANEs) across the United States, especially in medically underserved communities. The purpose of this study was to describe the lived experiences of nurses on their journey before and after becoming a SANE.

**Design:**

A qualitative descriptive research design was used in this study.

**Methods:**

A semistructured interview was conducted among 10 nurses who completed SANE training from a medically underserved community.

**Findings:**

Perceptions of being valued and supported and programme rigour influenced trainees' commitment to complete the SANE programme. Misconceptions about the role changed as trainees were immersed in the training. Being isolated and underutilized from the health care delivery system and the broader medico‐legal system were faced by SANEs after completing SANE training. Continuous use of telemedicine, continued funding for SANE training and greater emphasis on the legal aspect of the role during training are recommended by SANEs.

**Conclusion:**

Trainees' experiences during SANE training influence programme retention and completion. To fully build the capacity of SANEs in the community, an infrastructure and a system must be developed to embrace and integrate new SANEs into the broader medico‐legal system so they can perform in the role they are trained for.

**Implications:**

The study's findings have implications for policies in compelling medico‐legal and political systems to help build SANE capacity in the community, especially in medically underserved regions. Likewise, continuous input and involvement of trainees in the training implementation are critical to SANE programme retention.

**Impact:**

This study provides some of the lessons learned in the training and education of nurses to become SANEs, which could be helpful to clinical educators wanting to establish a SANE programme, as well as factors that can lead to the decline of SANE capacity after so much investment has been made to it.

**Summary**

What Does This Paper Contribute to the Broader Global Clinical Community?

- This paper presents some of the best practices and lessons learned in building the capacity of SANEs that are transferable to medically underserved and resource‐poor regions globally.

## 1 Introduction

Sexual assault is one of the most underreported violent crimes in the United States [1]. Sexual assault nurse examiners (SANEs) conduct a comprehensive medical forensic examination, which includes the evaluation and treatment of injuries, obtaining the medical‐forensic history, conducting the physical assessment, providing support and crisis intervention, offering prophylaxis against sexually transmitted infections, assessing pregnancy risk and offering treatment options and providing follow‐up referrals for medical and emotional needs for sexually assaulted victims [2, 3]. Because of SANEs' work, increased prosecutions were reported on perpetrators of crimes relating to sexual violence [4]. However, there is a shortage of SANEs across the United States, especially in medically underserved communities. In US border regions where this study was conducted, victims of sexual assault were being transferred to other facilities 150 miles away, causing treatment delays and further trauma to sexual assault victims. Thus, SANE capacity building in resource‐limited regions and understanding nurses' journey to becoming a SANE are critical.

Reports about barriers to becoming a SANE and the challenges faced while undergoing SANE training have been reported in the literature [5, 6, 7, 8]. Significant investments have been made to increase the capacity of SANES through grant funds to address the SANE shortage, especially in underserved regions [9]. Recent reports have been published about SANE training programmes implemented across the nation [6, 7, 10, 11, 12]. However, little has been reported about the journey nurses from medically underserved regions lived towards and after becoming a SANE. Thus, the purpose of this study was to describe the lived experiences of nurses during and after participating in a SANE training programme. This study is significant because the findings of this study could inform SANE clinical education administrators on curriculum development and the types of support SANE trainees need while in the SANE programme and after completing the programme. The lessons learned in this study could be helpful for clinical educators who are aiming to develop or enhance a SANE programme in resource‐limited settings. Likewise, the results of this study could inform on strategies that need to be in place before implementing a SANE programme and policies to compel the political and medico‐legal entities in the community to be more proactive and provide support in building SANE capacity in the community. By understanding the trainees' journey, the capacity of SANEs can be built and sustained, especially in medically underserved and resource‐limited regions.

## 2 Background

### 2.1 Barriers to Nurses

The literature is abundant, with reports indicating the barriers that prevent nurses from obtaining SANE certification. The significant barriers to becoming a SANE are the cost of training [13], lack of time and support from employers for nurses to obtain SANE certification [6, 14], lack of additional compensation afforded to nurses for performing SANE‐related duties [7], lack of full‐time employment for nurses to do only SANE duties [14] and lack of SANE preceptors to supervise trainees [14]. Nurses are hesitant to become a SANE due to their discomfort with the topic related to sexual assault and the psychological burden resulting from the victims' stories [15]. Due to the nature of their work, SANEs have a higher risk for compassion fatigue, emotional distress, burnout and vicarious trauma [7, 8, 15, 16, 17]. On‐call duties, emotional and psychological burdens and work‐life balance struggles contribute to nurses' reluctance to become a SANE [5]. Aside from the financial and time investment, preceptors, mentors and clinical placement sites to meet certification requirements are scarce [11]. Rural areas have low caseloads, making certification renewal difficult, resulting in skills and knowledge loss among newly certified SANEs [18, 19].

Despite such challenges, SANEs perceived their work as gratifying, knowing that they could help victims of sexual assault and that they could make a difference in their lives [5]. While the literature is clear about the barriers to becoming a SANE, there needs to be more information describing nurses' journey towards becoming a SANE and after becoming a SANE, especially among those who reside in resource‐limited regions. Understanding the education and training of the SANE workforce could provide information on the types of support they need. It could inform policy on how the community's local medico‐legal and political systems could contribute to building and sustaining SANE capacity.

## 3 The Study

### 3.1 Aim

The purpose of this study was to describe nurses' lived experiences on their journey to becoming a SANE nurse and the challenges they faced after obtaining SANE certification. For this study, SANE certification is defined as the attainment of Adult SANE Certification (CA‐SANE) and Pediatric SANE Certification (CP‐SANE) issued by the Office of the Attorney General (OAG) of the state of Texas.

## 4 Method

### 4.1 Study Design

A qualitative descriptive research design was used in this study. This approach is appropriate for understanding people's cultural or lived experiences in natural settings [20, 21]. A theoretical context was not the focus of this investigation but aimed to describe participants' experiences. Qualitative descriptive studies are the least ‘theoretical’ of all qualitative approaches [22, 23, 24]. The approach focuses on providing a straight description and comprehensive summary of the phenomenon of interest using participants' language and staying close to the data [21]. Individual virtual semistructured interviews were conducted among participants of a SANE training programme.

### 4.2 Setting and Participants

This study was conducted in one medically underserved border region between the United States and Mexico. The study participants were nurses in a SANE training programme from 2018 to 2023. We refer the reader to previously published papers about the SANE training programme [12, 19]. The SANEs were contacted by a graduate research assistant through texts and emails to participate in a semistructured interview through Microsoft Teams using an interview guide (Table 1). SANEs who have obtained certification from the OAG of Texas for 1 to 2 years were recruited. Ten SANEs agreed to be interviewed.

**TABLE 1:** Semistructured interview questions.

Tell me about the experience you received with the SANE Programme? Please elaborate on the education and training you received from the programme? Please describe the positive aspects of the training. Please describe the challenges you encountered while being enrolled in the SANE programme? Tell me about the experiences you experienced AFTER obtaining SANE Certification? Please describe the value of having SANEs in sexual assault response teams (SART). Tell me about your perceptions of the SANE Role? Describe what you think is the role of a SANE in this community? Tell me about your thoughts on the sustainability of SANEs and SANE infrastructure in this community? Describe your thoughts and perceptions about being a SANE before enrolling in this programme? Did your perceptions change over time? If yes, please describe how these may have changed? After completing the SANE training, describe your thoughts about being a SANE in this community?

### 4.3 Data Collection

The study team comprised the first and second author and a graduate research assistant. The first and second authors trained the programme's graduate research assistant and nonadministrator to conduct semistructured interviews. A nonadministrator was selected to conduct the interviews to avoid influencing the participants' responses. Informed consent was obtained prior to the interviews. Participant recruitment and interviews continued until data saturation. The first and second authors conducted preliminary assessments of the transcript for emerging themes even at the data collection stage. When it was determined that no new information emerged from each interview question item, it was determined that data saturation was reached [25]. The interviews were recorded and transcribed verbatim by a trained graduate research assistant. All interviews were conducted in English.

### 4.4 Data Processing and Analysis

Data were analysed using thematic analysis following the method proposed by Braun and Clarke [26]. Braun and Clarke's [26] thematic analysis was used to identify, analyse and report patterns in the data. This type of analysis has been used in conducting qualitative research in psychology, nursing, midwifery and others [27, 28, 29, 30]. Data analysis was conducted by hand in six steps:

1. This step involved reading and rereading the transcript to obtain familiarity and a broad understanding of the data.
2. Codes were generated manually after familiarization with the data. Codes are elements in the data that pertain to the research purpose. Data extracts or quotes were matched to each code.
3. The generated codes and their matching data extracts were sorted and examined for patterns or themes.
4. Similar themes were collapsed. Data extracts were re‐examined to determine the suitability of the identified theme. If data extracts did not match the theme, these were relocated to other themes, or a new theme was developed. Coding and recoding were ongoing until the data extracts, codes and themes fit together.
5. The essence of each theme was examined for its fit to the overall topic of investigation.
6. The relation among themes and interpretation of the ‘story’ between and among the themes was explored (Table 2).

**TABLE 2:** Themes.

**Themes**	**Participant**	**Codes**
Programme support	10, 7, 5, 4, 1, 3, 9, 8, 6	Programme staff support; arranged out‐of‐town clinical placements; instructors were knowledgeable and willing to train; flexibility and accommodation for trainees' busy schedule; financial assistance; administrative assistance with certification renewal.
Quality of SANE programme design	5, 4, 1, 2, 3, 10, 9, 8, 7, 6	Self‐paced online didactic; high quality education and training using patient models; assistance with clinical placements at trauma centres, gynaecology and paediatric sites; court observation and mock trials; SANE programme's collaboration with other clinical facilities.
Feeling valued and a sense of gratitude	5, 6, 7, 8	Inclusion of SANE trainees in stakeholder meetings and decision making; gratitude for the resources brought to the community.
**Challenges during SANE training**		
Lack of employer support for travel	6, 7, 1	Needing employer support to get the time off to travel to clinical sites; distant travels to clinical sites.
Lack of hospital SANE infrastructure	8, 7, 5, 2, 3, 10	Local hospitals do not have existing sexual assault protocols; lack of equipment for documentation such as a camera; no dedicated examination room for sexual assault victims; no formal on‐call list of SANEs; no SANE preceptors; ER physicians unfamiliar with forensic medical examination protocols; no SANE teams at local hospitals.
Lack of cases	10, 9, 3	Inability to examine cases on their own due to lack of local cases to get certified and recertified.
Lack of SANE preceptors	1, 7, 9, 5	No SANEs on call to supervise trainees.
Personal challenges	10, 7, 4, 2, 3, 6	Need to get comfortable with sensitive nature of the role especially listening to the victims' stories; time management and work‐life balance.
**Positive aspects after obtaining SANE certification**		
Rewarding feeling	2, 10, 9, 8, 7, 6, 5, 1, 3, 4	Rewarding feeling from being able to help and not send victims out to other facilities; great satisfaction with new skills set as a SANE; pride with new initials as a SANE.
Changes in role perceptions	6, 10, 2	Great appreciation of the programme; SANE role is a newfound passion.
Trauma‐informed care	2, 4, 3, 1, 8, 7, 3, 5	Learned to talk to victims without revictimizing them; learned empathy and compassion; emphasized individualized care; advocacy; ensure victims' safety and mental well‐being.
**Challenges after becoming a SANE**		
Lack of local preceptors	1, 4, 8	No local preceptors to get supervision.
Lack of local cases	9, 10, 8, 1, 5, 2	Not enough local cases; unable to perform examination on their own; no case reviews.
Lack of coordination with law enforcement	6, 3	Long wait for police department to come and pick up the rape kit; uncoordinated work with police.
Lack of SANE duty compensation	5, 1, 2	Volunteering hours as a SANE with no compensation; unpaid SANE service if done at a hospital wherein one is not currently employed.
Lack of integration in the medico‐legal system	10, 6, 3, 8, 2, 5, 7	Training and skills are not being utilized; needing feedback from law enforcement about how the evidence that was collected helped the case; community is unaware of the existence of SANEs; SANEs not included in the wider medico‐legal system in the community.
**Future directions**		
Need to get involved	8, 2, 5, 7, 6, 9, 10	Educate the community about the presence of SANEs in the community; work on SANE protocols; help establish a SART in the community.
Continue to build SANE capacity	7, 6, 5, 2, 10, 9, 3, 6, 8, 4	Continue funding support to train local nurses to become SANEs and prevent transfers of victims to distant facilities.
Build an infrastructure for a SANE team	10, 7, 6, 2, 9, 5, 2, 3	Build a SANE team that will cover 24/7 duties; build SART in the community.
Free‐standing SANE facility	2, 9, 7, 6, 4, 1, 3	A free‐standing SANE centre to decongest the ER and provide prompt care to victims.
Telemedicine access with SANE experts	7, 8, 5	Set up SANE experts on telemedicine.
Things to Improve in the SANE training programme	6, 5, 3	Case reviews: mock trials, expert witness simulation and feedback on collected evidence.

### 4.5 Ethical Considerations

Approval from the Texas A&M International Institutional Review Board (#2018‐09‐27) was obtained last January 25, 2023, before data collection was conducted. Participation was voluntary.

### 4.6 Rigour and Reflexivity

Using Braun and Clarke's [26] method of thematic analysis, the two authors independently reviewed the transcript to identify the preliminary themes. The themes were generated from the patterns of data extracts from the participant's narratives. If data extracts did not match the theme, these were relocated to other themes, or a new theme was developed. Peer discussions between the first and second authors were conducted to compare themes extracted from the individual analysis. Discrepancies in the themes were discussed until a consensus was reached between the authors. One of the authors is a Hispanic resident of the area, with insight and inside information regarding a border region's Hispanic culture and contexts. The other co‐author was of Asian descent. The diversity of the authors' cultural perspectives enabled rich discussions about the themes gathered in this study. The findings of the study were presented at the stakeholder meeting. Study participants, SANE trainees and community members attended this meeting. The purpose of the stakeholder meeting was to solicit input from stakeholders on ways to improve the SANE training programme and how it can best serve the community.

## 5 Findings

This study aimed to understand the lived experiences of nurses from medically underserved regions on their journey towards and after becoming a SANE. Among the 10 participants, four were certified for adult (CA‐SANE) and six were certified for paediatrics (CP‐SANE). The length of experience as a SANE ranged from 13 to 53 months; the average is 29 months or close to 2 ½ years. The average age of the participants was 41; four held a BSN degree, while six had a master's degree in nursing; 70% were employed at the hospital. The length of nursing experience ranged from 5 to 37 years (Table 3).

**TABLE 3:** Demographic profile of participants.

Certification type	SANE Experience in months	Ethnicity	Sex	Age	Education	Geographic location	Place of employment	Years of nursing experience	Area of experience
CA‐SANE	53	White	F	65	MSN	Local	University	37	Medical surgical nursing
CP‐SANE	27	Hispanic	F	36	MSN	Local	Hospital	6	ICU
CP‐SANE	26	Hispanic	M	27	BSN	Local	Hospital	5	ER/ICU
CA‐SANE	42	Hispanic	F	41	BSN	Local	Hospital	8	ER/ICU
CP‐SANE	16	Hispanic	F	47	MSN	Local	University	23	NICU/paediatrics
CA‐SANE	38	Hispanic	F	34	MSN	Local	Hospital	10	ICU/medical surgical nursing
CP‐SANE	13	Hispanic	M	28	BSN	Local	Hospital	5	ICU/medical surgical nursing
CP‐SANE	17	Hispanic	M	62	MSN	Local	Clinic	37	Paediatric
CA‐SANE	38	Hispanic	F	38	MSN	Local	Hospital	15	Medical surgical nursing
CP‐SANE	20	Hispanic	M	36	BSN	Local	Hospital	13	Medical surgical nursing

### 5.1 Positive Aspects of the SANE Training Programme

Overall, the participants verbalized positive experiences with the SANE training programme. Programme support, quality of the programme design, flexibility in the training schedule, skills development and collaboration were some of the positive experiences expressed by SANEs about the training. Being valued as a trainee and having excellent communication with programme staff during training were critical to programme retention. A sense of gratitude was expressed in the opportunities the grant provided for the participants and the community (Table 4).

**TABLE 4:** Factors impacting SANE trainee success.

Factors associated with SANE trainee completion
Programme staff support
Quality and rigour of the programme
Being valued in the whole SANE training process
Excellent communication between SANE Programme staff and trainee
Flexibility of didactic and training schedule
Assistance in clinical placement to a variety of clinical sites
Tuition and travel stipends
Administrative assistance in the submission and renewal of certification
Prompt feedback for expert SANE preceptors and live patient actors

#### 5.1.1 Programme Support

The support, assistance and attention from programme staff to trainees were critical to positive experiences in the SANE training programme. Because the trainees already have a busy work and life schedule, the SANE training components were organized to consider these. For example, the online SANE didactic was self‐paced. Tuition and travel stipends were provided to offset the financial burden of SANE training [12]. Administrative assistance was provided upon submitting certification applications to the certifying body. Because of the attention and assistance provided, the participants felt valued in the training process, which led to gratitude for the resources.

The program staff was available … she's great. She made this experience really, really easy. She guides everyone through everything and like I said, she's always available, she answers really fast. (Participant 10)

Program staff helped me a lot on the clinical practicum that I needed for my certification. I felt confident with the training experience I went, and with the places that the SANE Program was collaborating with. (Participant 7)

#### 5.1.2 Quality of SANE Programme Design

The participants perceived that the SANE programme was of high quality, rigorous and worth the time they invested in travel, especially to out‐of‐town clinical sites, to obtain supervised hands‐on experiences. Exposure to various clinical sites provided an understanding of how a SANE infrastructure is set up in metropolitan hospitals. Immediate feedback from multiple preceptors across clinical sites, instructors and live patient actors helped trainees correct deficiencies in competency, which helped build self‐confidence.

I think that the SANE Program structure is simple. I felt pretty confident with the training I received especially with the places I went to obtain hands‐on experience. (Participant 5)

It's top‐of‐the‐line education, the online programs that the SANE Program provided for us, the person‐to‐person experience that we did in training and for myself in Houston, it was really good. (Participant 4)

#### 5.1.3 Feeling Valued and a Sense of Gratitude

The feeling of being valued was important to participants' remaining enrolled in the programme. Excellent communication from SANE programme staff while also including trainees in the quality improvement of the SANE programme made the participants feel valued in the training process. The trainees' input for programme improvement solicited during stakeholder meetings was critical to programme buy‐in.

The Program Manager was instrumental to keeping this program going, keeping us up, keeping us informed, and including us in meetings that involved community stakeholders to give an input. That really has helped us feel valued and contribute to the success for the program. (Participant 5)

I was given knowledge and given an opportunity to be able to be part of this, so I am very thankful for that. (Participant 6)

### 5.2 Challenges Encountered During the SANE Training

Although the participants expressed positive experiences while undergoing SANE training, they also encountered many challenges. These challenges included lack of employer support for travel to complete SANE certification requirements, lack of local hospital infrastructure and protocols for sexual assault, lack of cases in the community and lack of local SANE preceptors to guide trainees during clinical rotation and personal challenges.

#### 5.2.1 Lack of Employer Support for Travel

The participants verbalized the travel limitations imposed on them by their employers. It is possible that such limitations could be due to the nursing shortage already existing in a medically underserved region. Finding coverage for open shifts was potentially a challenge for hospital administration, leading to nurses' travel and time‐off restrictions.

It is a lengthy process to get everything going from the clearance of our employer to getting the time off to go do our clinicals. (Participant 6)

I believe some of the challenges was mainly the travelling. The trips that we had to do to get certified; it was difficult to get clearance from the hospital. We had to go to Houston, Edinburg, and San Antonio. (Participant 7)

#### 5.2.2 Lack of Hospital SANE Infrastructure

All participants were placed in out‐of‐town clinical sites because the local hospitals did not have the infrastructure to handle sexual assaults such as standardized protocols and equipment to conduct medical forensic examinations. A SANE team in local hospitals has not been established; thus, response to sexual assault cases was unstructured and uncoordinated.

I think the biggest challenge was the lack of structure when it came to the actual settings, locally. There is none. (Participant 5)

The local hospitals are not up to date with the protocols. They do not have a dedicated private room for the patient to be examined. A room with not too much noise, and patients going in and out. (Participant 8)

#### 5.2.3 Lack of Local Cases

Because of the lack of SANEs and hospital equipment for medical forensic examination, sexual assault victims were being transferred out to the nearest facility more than 150 miles away. This resulted in the lack of cases where SANE trainees could not complete certification requirements within the community. Consequently, SANE trainees needed to be placed in out‐of‐town facilities for hands‐on clinical experiences.

The lack of local cases made completing the certification and recertification requirements a challenge prompting the participants to go out town. (Participant 10)

It's hard to get pediatric cases done, so it's not so much of the difficulty of performing the exam. It was sometimes like an ebb and flow. Sometimes there's an influx of cases and then I'm unfortunately not available. (Participant 3)

#### 5.2.4 Lack of Local SANE Preceptors

Due to the lack of SANEs in the area, there were no preceptors available to guide trainees during the conduct of a medical forensic examination. Although several SANEs have already received certification since the inception of the SANE programme in 2018, the local hospitals do not have a structured SANE team and a SANE on‐call list. Thus, when a SANE trainee was called upon for a sexual case, no SANE preceptor was available to supervise the clinical experience.

Here locally, one of the challenges was mainly get people available to help you out late at night. If there was a case at the at the hospitalcc. (Participant 7)

I was told there was a schedule, there was a backup person, but that person was never often available. Because of the timing and everything going on you know my recertification may be difficult to actually be accomplished. (Participant 1)

#### 5.2.5 Personal Challenges

Although travel expenses were reimbursed and stipends were provided, out‐of‐town travel to clinical sites posed a challenge to the participants. It seems that the travel was a burden to the trainees' work‐life balance. On the other hand, the personal stories of the sexual assault victims resulted in some emotional and psychological burden among trainees.

I believe some of the challenges was mainly the travelling, you know, the trips that we had to do to get certified. (Participant 7)

It's sensitive things, you know, they are sensitive things it's not just like any regular patient. I guess once you get called it's about being comfortable with what you hear from a patient on what had happened to them or a mother talking about her kid on what had happened to him/her. (Participant 10)

### 5.3 Positive Outcomes After Obtaining the SANE Certification

Before enrolling in the SANE programme, the participants had misconceptions about the SANE training and the role SANEs play in the care of sexual assault victims. However, these misconceptions slowly changed as soon as they were immersed in the training. The participants realized that participating in the SANE training was the right decision and that their role was not just collecting evidence. They learned to embrace that their role is to help sexual assault victims' healing process.

#### 5.3.1 Rewarding Feeling

The participants verbalized a rewarding feeling after enrolling in the SANE training programme. There was a sense of accomplishment; the out‐of‐town travel for clinicals was worth the time invested. The added credentials after their name and the ability to be of service to sexual assault victims, which was lacking before in the community, provided a sense of accomplishment and pride among the SANEs.

It asks a lot; it's a tough subject but the benefits are rewarding. What you feel, what you get to do for these victims or these survivors it's very rewarding. (Participant 2)

It feels great to carry those initials in the badge. It feels great that I can help somebody and make a difference in their lives; make a terrible moment or a terrible event less terrible and make somebody not feel alone. (Participant 10)

#### 5.3.2 Changes in Role Perceptions

Before enrolling in the SANE training programme, some misconceptions were that obtaining a certification would be impossible and that the training components were complex. However, this changed over time from the assistance afforded to the trainees during training and once they understood the role of SANEs.

To feel comfortable with what the job requires because it is sensitive things; but you know what, this is a job that needs to be done, and we need nurses who are capable of doing these kinds of exams for the community. The misconception was that ‘that it's very hard’ but it's very rewarding. (Participant 6)

#### 5.3.3 Trauma‐Informed Care

SANEs perceive that therapeutic communication is a vital aspect in forensic nursing care to start the healing process among sexual assault victims. Trainees reflected that their most crucial duty is to communicate with sensitivity and not induce additional trauma among victims.

I think the biggest aspect I took was how to talk to the survivors. Evidence collection was important, but as nurses we know how to properly collect, so I do not feel that was a big one, but mainly how to speak to our survivors. You have to try to find the right words to use to get them to tell their story or their report. (Participant 2)

Provide trauma‐informed care for our victims making sure that they start their healing process. I feel it is more of healing them knowing that someone did care or letting them know their options. (Participant 2)

### 5.4 Challenges Faced After Becoming a SANE

The challenges SANEs faced after obtaining SANE certification, especially during certification renewal, were like those encountered when seeking SANE certification for the first time. The lack of local SANE preceptors, cases, hospital coordination with law enforcement and integration of the SANEs into the local medico‐legal system prevented SANEs from truly performing their role. Due to local hospital policies, SANEs who were not facility employees could not take SANE on‐call duties. This resulted in SANEs being unable to practice independently, which led to the loss of knowledge and skills and the expiration of SANE certifications, further depleting a promising SANE capacity in the community.

#### 5.4.1 Lack of Local Preceptors

I was told there was a schedule, there was a backup, but that person was never really often available. (Participant 1)

I'm not an employee of either facility, so I have not been able to truly practice. (Participant 4)

#### 5.4.2 Lack of Local Cases

Not being to perform a case on my own … on the days I am on call it's like nothing happens. For recertification in the future, I need to have cases done; we do not have case reviews. (Participant 9)

I have not been able to perform any case on my own. I did the live models; I did the cases out of town, but I was under somebody … but by self myself after certification, no. (Participant 10)

#### 5.4.3 Lack of Coordination With Law Enforcement

Because local hospitals do not have standardized SANE protocols, the response to sexual assault cases has been uncoordinated and disjointed. The uncoordinated efforts resulted in delays in providing services to sexual assault victims. There seems to be a lack of communication between SANEs and local enforcement in responding to sexual assault cases.

We do not have a lock box, so we wait for law enforcement to pick up the kit … and they take a bit longer to pick it up … we need to work together. (Participant 6)

Unfortunately, we do not get feedback from our law enforcement to let us know how our evidence helped bring someone to justice or to just let us know if we did a good job. I think maybe just a little bit more of a closed loop communication like … hey, on that case you did two months ago, this is what happened. (Participant 3)

#### 5.4.4 Lack of SANE Duty Compensation

No pay structure or incentives were available for SANEs who take on‐call duties at facilities where they are not employed. The lack of compensation is discouraging, removing motivation to renew SANE certification.

If you are not employed by one of the hospitals, you are not getting paid for it because you have to be an employee of the hospital in order for them to pay you. I'm not employed by either facility, so that also brings up the legal part of what we do. I have not been able to truly practice. (Participant 5)

#### 5.4.5 Lack of Integration Into the Medico‐Legal System

After receiving training and certification, the local medico‐legal system and the wider community were not ready to integrate the SANEs. A structured SANE on‐call list was not set up at local hospitals, resulting in transfers of sexual assault victims to other facilities. Standardized protocols detailing the coordination of local law enforcement, advocacy groups and resources in handling sexual assaults did not exist. The space and equipment needed to conduct medical forensic examinations were unavailable at local hospital facilities.

I think the biggest challenge was the lack of structure when it came to the actual settings, I asked them where's the camera? Where's this? Where's that? How do we upload our pictures? I did speak to one of the physicians one night. He (ER doctor) himself too, felt a little uncertain of the process and what to order. So, I kind of guided him on the protocol I learned from training.(Participant 5)

Right now, we are working on getting some protocols installed in our facilities. There are no set protocols for our local hospitals like in Houston or Edinburgh. Over there, they have an office in the hospital for SANE nurses. (Participant 7)

### 5.5 Perceptions About Future Directions

After having experienced all the constraints during and after completing the training, SANEs felt that they needed to be more proactive, reach out to the community and make themselves known to the local medico‐legal system and the wider community. Historically, there was only one certified SANE in the community until 2018, when the grant funding was received, and SANE training began. Thus, the lack of awareness of SANEs in the community must be addressed through community outreach to local policymakers.

#### 5.5.1 Need to Get Involved

I think we need to start to try to talk to politicians to see how we can do more. Maybe talking to the district attorney to see if he can help us out. We have to put ourselves on the table, talk to the local community, local agencies, and authorities so we can start working on the protocols so that we can have them available for us to use. This way there will always be a SANE, you know, available to you locally. Right now, the way it's set up … it's very challenging especially if you do not know which contact to go to or if you do not have any contacts. (Participant 8)

#### 5.5.2 Continue to Build SANE Capacity

The SANEs recommended continued funding to train nurses in the region to become SANEs. Having more SANEs could help offset long waits for victims, cover on‐call duties, prevent burnout and maintain SANE capacity.

I hope funding continues to support nurses who want to join this program. And I'm sure that over time in a few years from today, there will be enough staff to be on call 24/7 on both hospitals and attend to the needs of the victims. (Participant 10)

We do not have that many pediatric SANE nurses. The more nurses that we can have available for our communities, the better the outcome for the victims. (Participant 6)

#### 5.5.3 Build an Infrastructure for a SANE Team

A SANE infrastructure at the local hospitals needs to be established. A dedicated SANE team needs to be created to develop sexual assault protocols and coordinate with local agencies to respond to sexual assault cases. An on‐call schedule needs to be established to allow trainees to conduct precepted medical forensic examinations even if they are not facility employees to continue building the SANE workforce in the community.

Start working on protocols, so I think it's essential to have a nurse in that task force, you know, which I did not see when they were promoting awareness of sexual assault in this town. (Participant 8)

Also, hospitals have not allowed nurses not employed at the facility to come in and conduct an examination even though an Affiliation Agreement exists. (Participant 5)

#### 5.5.4 Free‐Standing SANE Facility

The SANEs recommended that a free‐standing SANE facility for sexual assault victims who are clinically stable be established in the community due to the overcrowding in the local emergency rooms. The facility would prevent long waits and prompt care for victims. Emergency room resources could then be focused on patients who have life‐threatening conditions.

The ER is the only route right now where our victims can go to, but in all honesty, sometimes the environment is not the best and can be traumatizing to our victim. Not everybody is willing to go to the ER for other reasons. I know due to COVID there are still some individuals that do not want to step foot into the hospital. So having that outside facility or center could really benefit the victims to provide them comfort, and serve as an educational or even as a resource for victims, kind of like a safe haven for our victims. (Participant 6)

#### 5.5.5 Telemedicine Access With SANE Experts

The SANEs recommended continuing the telemedicine set‐up with SANE experts across the state that was initially established through the grant. Through telemedicine, medical forensic examinations were conducted, which prevented transfers of victims to nearby facilities. This also enabled SANE trainees to complete their certification requirements locally.

I did a few cases here locally but that was in consultation and telecommunication with a SANE nurse from another hospital to guide me through the exam. It was a challenge because you have to find your preceptor to guide you (Participant 5).

#### 5.5.6 Things to Improve in the SANE Training Programme

When asked about the improvements that could be made to the SANE programme, the participants recommended more hours be allocated for courtroom observation and mock trials. Simulations relating to expert witness testimony and case reviews are needed to become more familiar with the legal aspect of the role.

Us wanting to continue our education by doing case reviews. I think that's what's really helped me at least continue and prepare for my SANE role. The online didactic, the court observations, the case reviews really did help bring a perspective of what to look out for, what to be ready for. Also, more expert witness simulation would help us out a little bit more(Participant 6).

## 6 Discussion

This study examined nurses' lived experiences on their journey towards becoming SANE nurses and the challenges they faced after obtaining SANE certification. The current findings corroborate past literature citing the challenges nurses face towards becoming a SANE [5, 6, 7, 8, 14, 15] but also add to the current knowledge by presenting the challenges SANEs face after obtaining certification and integrating into the community's medico‐legal system. The current findings describe how hospital policies and the local political landscape can sometimes hinder building SANE capacity.

Programme staff support, quality of the SANE training programme, support and inclusivity are factors that influence participant buy‐in and, therefore, retention in a SANE training programme. Programme retention is influenced by resources and support afforded to trainees. Assistance in finding preceptors and clinical placements and maximum flexibility in didactic and clinical training schedules are critical to programme completion and retention. Past studies indicate SANE trainees' challenge in finding preceptors to complete the cases needed for certification [11, 14, 18]. Lastly, obtaining a SANE certification must add value to a nurse's professional career and be worth their time and investment. A sense of fulfillment, personal satisfaction, purpose and self‐pride drive nurses to become and remain a SANE. The role is no longer viewed as a technical or procedural endeavour but more of a humanistic undertaking and an advocacy, ensuring that victims receive proper care and transitions towards healing.

The challenges encountered by SANEs during training and at the certification renewal period were similar. These included the lack of preceptors, cases in the local community and infrastructure from local hospitals, particularly standardized SANE protocols. In addition, local hospitals' organizational policies were counter to building a capacity of SANEs in the community. Despite established affiliation agreements, SANE trainees who were nonemployees of the facility could not take part in SANE on‐call duties. The lack of financial incentives for SANEs and the lack of integration of the latter into the wider medico‐legal community resulted in diminishing motivation to maintain and renew certification. These nurses found themselves isolated and underutilized despite the rigorous training they underwent to become a SANE.

The local hospitals were not quite ready to integrate the SANEs into the healthcare delivery system, as evidenced by the lack of sexual assault protocols, medical equipment and infrastructure to address sexual assault. More importantly, an active local sexual assault response team (SART) has not been established despite the presence of new SANEs in the community and the legislative mandate, Senate Bill 476 [31]. The underutilization of SANEs and lack of infrastructure resulted in the continued transfer of sexual assault victims to other facilities.

To achieve SANEs' integration into the medico‐legal system, stakeholders, including the political, legal, nursing, medical and hospital community, need to be involved in the planning and developing a SANE programme. Clinical educators need to conduct a needs assessment before programme implementation to explore the existence or absence of an infrastructure to support a SANE programme. The appraisal will include exploring hospital policies that are supportive or not for the implementation and sustainability of a SANE programme in the community. These may consist of the infrastructure, including standardized sexual assault protocols, space, medical forensic equipment, mentors and support from ER medical staff, hospital policies to accept SANE trainees for clinical rotations and incentivization of nurses after completing and obtaining SANE certification. An assessment of the above will promote the sustainability of SANE capacity in the community. Moreover, strategic planning, which will include the medico‐legal community, is critical in exploring the existence or the lack of resources in the community concerning the care of victims from sex violent crimes. The agenda of the medico‐legal community that will be working with the future SANEs in the community needs to be explored and considered to promote community buy‐in. Gathering input from the medico‐legal community on measuring programme success will create accountability and responsibility on how the latter can contribute to the success and sustain SANE capacity. Strategic planning will detail specific timelines and outcome measures, key programme milestones will be monitored and delays in achieving outcomes will be documented and presented in stakeholder meetings. By conducting strategic planning, potential constraints of programme implementation could be identified, which may prevent the challenges encountered in the current study. Identifying and establishing a SART at this stage is critical so that nurses who complete the SANE training will seamlessly be integrated into the team and function at their full potential. Finally, the creation of SART protocols will promote a coordinated response to sexual assaults in the community.

To sustain SANE capacity, especially in medically underserved regions, continued funding, recruitment, education and training of nurses are needed. With the strong career market for nurses outside the SANE role, continued capacity building is needed to establish teams to cover on‐call duties, eliminate long waits and transfers of victims to other facilities, prevent burnout and develop preceptors and mentors. Burnout has been reported as one reason nurses leave the SANE role [8, 15, 16]. A free‐standing SANE facility dedicated to sexual assault victims who do not have life‐threatening conditions is needed in medically underserved communities. Reimbursement policies for the medical forensic examinations conducted in free‐standing SANE centres must be established to encourage these centres to remain open and provide such a service. Such will help mitigate the long waits in the emergency room and provide prompt care to victims. In a medically underserved region, patients tend to use the emergency rooms for nonemergent needs, resulting in overcrowding, long waits and poor care for sexually assaulted victims [32, 33]. Finally, telemedicine needs to continue to connect SANEs with other SANE experts from other facilities across the state. Getting immediate guidance from SANE experts will lead to prompt care of sexual assault victims.

## 7 Conclusion

A positive journey towards becoming a SANE and after becoming a SANE is critical to sustaining SANE capacity. Participants stay committed to SANE training programmes when they experience support, assistance, flexibility, quality and rigour and an added value of the endeavour to one's professional career. Integrating SANEs into the broader medico‐legal infrastructure and allowing them to perform their role to its fullest will motivate these nurses to maintain their certification and remain in this nursing field. Therefore, barriers that prevent nurses from fully performing the SANE role need to be recognized and addressed, otherwise, the community will see itself at the same spot where it started—the absence of SANEs. Establishing a community of practice may be one way to promote community awareness about SANEs and collaboration across all stakeholders involved in sexual assault response, such as law enforcement, local district attorneys, hospital facilities, advocacy groups and others.

The current study contributes to the current knowledge by showing practices that can contribute to the success of SANE training programmes and factors that can lead to the decline of SANE capacity after heavy investments have been made in building it. The results of this study could inform clinical educators globally who plan to establish a SANE programme in their communities, establish policies to compel local hospitals and the local medico‐legal and political systems to be more involved and engaged in building SANE capacity in the community and demonstrate a more proactive approach in responding to sexual assaults, especially in border regions where human trafficking are often reported [34, 35, 36]. SANE capacity building entails more than just trainees' successful attainment of a certificate but the actual integration of these nurses into the system and the community so they can perform their role. SANEs need to thrive in their role and in the wider medico‐legal infrastructure for the capacity of the community to endure over time. Success in SANE capacity building needs political will and buy‐in from significant community stakeholders, not just efforts from SANE training programmes and federal funding agencies. SANE capacity building is similar to a three‐layered concentric circle: the SANE trainee at the inner core, the SANE training programme on the second layer and the broader medico‐legal political system on the third layer. These three‐layered concentric circles need to be tight and should inform one another so that SANE capacity in the community will endure.

The findings are limited due to the sample interviewed in terms of their age, education, years of nursing experience and the geographic location of this study. Participants who did not complete the SANE programme may have a different journey and experience. Likewise, SANE trainees from other programmes and geographic locations may have different experiences before and after becoming a SANE. Finally, the participants interviewed in this study received support, stipends and incentives while enrolled in a SANE training programme. SANE trainees enrolled in unfunded SANE programmes may have different experiences. We recommend future research to explore interventions to mitigate barriers identified in the current study, comparative studies of SANE programmes across diverse geographic locations, evaluation of the long‐term impacts of free‐standing SANE facilities and improved integration of SANEs in the medico‐legal system on building SANE capacity in the community.

## Author Contributions

**Marivic B. Torregosa:** conceptualization, data curation, formal analysis, funding acquisition, investigation, methodology, project administration, resources, software, supervision, validation, visualization, writing – original draft, writing – review and editing. **Maria Del Rosario Benavides:** conceptualization, data curation, formal analysis, investigation, methodology, project administration, validation, visualization, writing – review and editing.

## Conflicts of Interest

The authors declare no conflicts of interest.

## Data Availability

The data that support the findings of this study are available from the corresponding author upon reasonable request.
